# Optimization of Visual Information Presentation for Visual Prosthesis

**DOI:** 10.1155/2018/3198342

**Published:** 2018-03-14

**Authors:** Fei Guo, Yuan Yang, Yong Gao

**Affiliations:** Xi'an University of Technology, School of Electronics Engineering, No. 5 South Jinhua Road, Xi'an 710048, China

## Abstract

Visual prosthesis applying electrical stimulation to restore visual function for the blind has promising prospects. However, due to the low resolution, limited visual field, and the low dynamic range of the visual perception, huge loss of information occurred when presenting daily scenes. The ability of object recognition in real-life scenarios is severely restricted for prosthetic users. To overcome the limitations, optimizing the visual information in the simulated prosthetic vision has been the focus of research. This paper proposes two image processing strategies based on a salient object detection technique. The two processing strategies enable the prosthetic implants to focus on the object of interest and suppress the background clutter. Psychophysical experiments show that techniques such as foreground zooming with background clutter removal and foreground edge detection with background reduction have positive impacts on the task of object recognition in simulated prosthetic vision. By using edge detection and zooming technique, the two processing strategies significantly improve the recognition accuracy of objects. We can conclude that the visual prosthesis using our proposed strategy can assist the blind to improve their ability to recognize objects. The results will provide effective solutions for the further development of visual prosthesis.

## 1. Introduction

Globally, around 45 million people suffer from blindness caused due to eye diseases or uncorrected refractive errors. Although much progress has been made to rectify visual impairments, there is still no effective treatment for blindness [1]. In recent years, implantable electronic devices (i.e., visual prosthesis) have been proposed as a viable solution to restore partial vision for the blind [2]. Many visual prosthetic systems such as the “Argus II” and the “Alpha IMS” have received clinical approval in the US and Europe. Visual prosthesis, often known as bionic eye, captures scenes using a video camera and then converts the information into low resolution electronic streams which stimulate the electrodes implanted in the vision processing center of the brain. The microcurrent stimulator generates electrical stimulation which is transmitted to the optic nerve. This excites neurons to generate phosphenes. The generation of these phosphenes in the visual cortex can be used to restore vision and give the blind the ability to recognize objects. Currently, due to technical limitations, the number of arrays in the stimulation electrodes is still very limited. The current electrode number of the Argus II retinal implant is 60 (10*∗*6). This number is expected to rise to about 1000 electrodes in the upcoming versions [3]. The Alpha IMS has 1500 electrodes [4]. Compared with the number of about 130 million sensing cells and 1.3 million ganglion cells in the normal human visual system, the limited number of electrodes can only elicit visual perceptions with low resolution.

To overcome the limited visual perception, researchers have tried to optimize the image presentation to deliver the effective visual information in the simulated prosthetic vision. Research groups have evaluated different approaches to improve the performance of the methods to optimize the visual information presentation. Boyle et al. [5] adopted two traditional processing methods (inverse contrast and edge detection) and two image presentation techniques (distance mapping and importance mapping) to evaluate the subject perceptions under simulated prosthetic vision with different resolutions and gray scales. Van Rheede et al. [6] proposed three image presentation methods (Full-Field Presentation, Region of Interest (ROI), and Fish Eye) based on retinal prosthetic vision. Results showed that the Region of Interest and fish eye methods increased the visual acuity of the prosthetic device user to produce favorable results during the static observation tasks. The Full-Field Presentation method performs better in visual tasks that need external environmental information. Zhao et al. [7] studied the minimum information requirement of simulated prosthetic vision aimed at solving the task of object and scene recognition. Lu et al. [8] proposed the projection and nearest neighbor search methods to optimize the presentation of Chinese characters and paragraphs. Results showed that the two optimized strategies increased the recognition of Chinese characters and the user's ability to read. Jung et al. [9] adopted an active confocal imaging system based on the light-field technology. The system was able to help prosthetic users focus on the object of interest while suppressing interference from background clutter. Wang et al. [10] proposed two image presentation strategies based on the background subtraction technique to segment moving objects from daily scenes to optimize the presentation in simulated prosthetic vision. Results from their research demonstrated that the adopted image processing strategies increased the recognition and response accuracy in low resolution. Parikh et al. [11] proposed image processing strategies based on improved Itti saliency detection model, respectively. Their results demonstrated that the saliency map can provide clues for searching and performing tasks for users with visual prosthesis. Wang et al. [12] and Li et al. [13] proposed two image processing strategies based on improved Itti and GBVS model to optimize the presentation in simulated prosthetic vision, respectively. Their results demonstrated that the use of the saliency segmentation method and image processing strategies can automatically extract and enhance foreground objects and significantly improve object recognition performance towards recipients with a high-density implant.

In terms of image processing strategy based on the saliency detection, most of the studies use the biologically plausible saliency visual model to extract the foreground objects. These sophisticated methods have low accuracy and high complexity. This leads to the segmentation being more complex (using “GrabCut” segmentation). Li et al. [14] proposed a real-time image processing strategy based on a novel bottom-up saliency detection algorithm. Their results demonstrated the effectiveness of adopting the novel saliency detection algorithm to improve the processing efficiency of strategy and enhance foreground object in a scene. Therefore, in this paper, to enhance the perception of salient objects in general dynamic scenes and improve the strategy processing efficiency, we proposed a saliency detection model based on the manifold ranking with an adaptive-threshold segmentation method and two image optimization strategies based on the saliency detection model. We perform experiments to test the effectiveness of the optimization strategies and evaluate the perception of daily scenes. The results demonstrated that the adopted saliency detection model has the advantage in terms of accuracy and speed over other methods, and our proposed strategies are able to improve the perception in daily life for the recognition of objects.

## 2. Image Processing Strategies Based on Salient Object Detection Model

**Figure 1:**
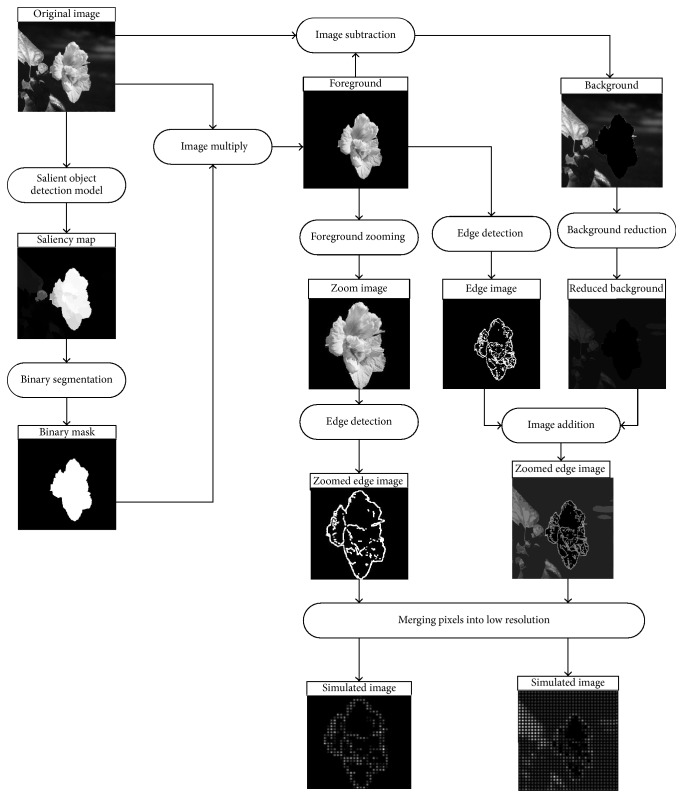
Schematic diagram of the two image processing strategies.

The image processing stage in visual prosthesis aims to set the resolution of original image corresponding to the number of stimulating electrodes and is called Lowering Resolution with Gaussian Dot (LRG). The limited number of electrodes in visual prosthesis can lead to huge loss of information when presenting the daily scenes. This severely restricts the ability of prosthetic implant to recognize objects in daily life. Increasing the contrast between the foreground region and the background region in real-life scenes can optimize the visual information presentation in simulated vision. Therefore, automatically detecting the main objects and precisely separating the objects from the scenes are needed first. In this paper, we define salient object as the main object and segment it as foreground. In Figure 1, we applied a salient object detection model for foreground extraction and combine it with a segmentation method for mask generation of salient objects. Furthermore, we proposed two optimized processing strategies to optimize the visual information presentation in simulated vision. They are called “Foreground Edge Detection and Background Reduction (FEBR)” and “Foreground Zoom and Edge Detection (FZE).” Finally, the processed images are presented in the simulated vision after the process of LRG.

### 2.1. Salient Object Detection

The extraction of the ROI region is based on the saliency detection technique. The saliency detection models are based on the visual attention mechanism and are used to extract the salient features to generate the saliency map. Common models such as the Itti and the GBVS are widely used in the field of visual prosthesis [12, 13]. However, the saliency map detected by the common model is discrete region [15, 16]. This makes the segmentation more complex. To address this limitation, we applied a two-stage salient object detection model based on manifold ranking. This model makes full use of the intrinsic manifold structure of the images to uniformly highlight the target and effectively compress the background [17]. Meanwhile, the model carries out a single inverse matrix operation and takes the super-pixel as the basic processing unit. This allows the model to gain in terms of accuracy and speed over other models.

The salient object detection model is based on the manifold ranking. The manifold ranking method proposed by Zhou et al. uses the intrinsic manifold structure of a data for graph labeling [18]. Let *X* = {*x*_1_,…, *x*_*i*_,…, *x*_*n*_} ∈ *R*^*m*×*n*^ be a dataset which consists of the queries and the data needs to be ranked. Let *f* : *x* → *R*^*n*^ be a ranking function, where each *f*_*i*_ is the ranking value of *X*_*i*_. This can be seen as a vector *f* = [*f*_1_,…, *f*_*n*_]^*T*^. Let *y* = [*y*_1_,…, *y*_*n*_] be an indicator vector. We have *y*_*i*_ = 1 if *X*_*i*_ is query; otherwise *y*_*i*_ = 0. A graph model *G*(*V*, *E*) is defined on the dataset, where the nodes *V* correspond to the dataset *X* and an affinity matrix *W* = [*w*_*ij*_]_*m*×*n*_ weighted the edges *E*. Given the graph *G*, the degree matrix is *D* = diag{*d*_11_,…, *d*_*nn*_}, where *d* = ∑_*j*_*w*_*ij*_. The ranking function can be computed as

(1) $$\begin{array}{c} f^{*}=\left(\;1-\alpha \;\right){\left(\;D-\alpha W\;\right)}^{-1}y, \end{array}$$

where *D* is the degree matrix, *W* is the affinity matrix, *y* is the indictor vector, and *α* is the parameter which controls the initial ranking value and the neighborhood propagation for the final ranking value.

**Figure 2:**
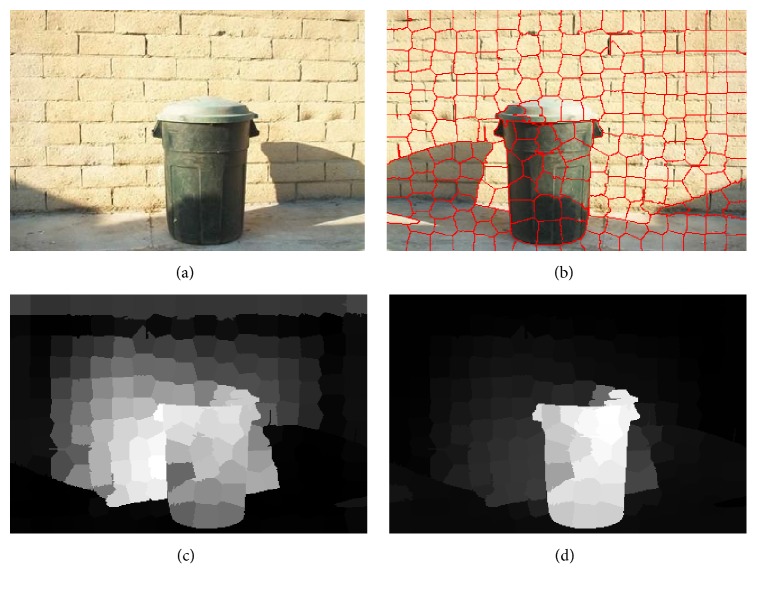
Diagram of the main step in the salient object detection model: (a) original image, (b) superpixels, (c) saliency map in the first stage, and (d) final saliency map.

In this model, the images are segmented into superpixels firstly by SLIC [19]. The saliency detection is as a manifold ranking problem and the saliency is measured by the ranking value of superpixels. Then, building a graph *G*(*V*, *E*), the affinity matrix *W* and degree matrix *D* can be computed. After that, a two-stage scheme is adopted in this model. In the first stage, this model uses the nodes on each side of images as labeled background queries based on boundary prior. Once the labeled queries are given, we can get the indictor vector *y* and compute the nodes saliency value by (1). We can get the four saliency maps using nodes of the top, bottom, left, and right side as queries by (2). The four saliency maps are fused to generate the saliency map *S*(*i*) in the first stage by (3). In the second stage, binary segmentation is applied on the saliency map *S*(*i*) and the foreground nodes set as salient queries. The saliency of each node is computed by (1) and the final map *S*_*f*_(*i*) is computed by (4).  Figure 2 shows the results of the main step in the saliency detection model

(2) $$\begin{array}{c} S_{i}\left(\;i\;\right)=1-\overset{-}{f^{*}}\left(\;i\;\right), \end{array}$$

where $\overset{-}{f^{*}}(i)$ is the normalized ranking vector *f*^*∗*^

(3) $$\begin{array}{c} S\left(\;i\;\right)=S_{T}\left(\;i\;\right)\times S_{D}\left(\;i\;\right)\times S_{L}\left(\;i\;\right)\times S_{R}\left(\;i\;\right), \end{array}$$

where *S*_T_(*i*) is the saliency map using nodes on top boundary as queries, *S*_D_(*i*) is the down-side saliency map, *S*_L_(*i*) is the left-side saliency map, and *S*_R_(*i*) is the right-side saliency map

(4) $$\begin{array}{c} S_{f}\left(\;i\;\right)=\bar{f^{*}}\left(\;i\;\right), \end{array}$$

where $\overset{-}{f^{*}}(i)$ is the normalized ranking vector of *f*^*∗*^.

### 2.2. Image Segmentation

The salient object detection model based on the manifold ranking outperforms other models in detecting salient objects. However, due to the presence of illumination and complex background, incorrectly classified pixels may be generated. Using the single-threshold in the saliency map segmentation cannot obtain the precise binary mask of objects. This reduces the accuracy of foreground extraction and affects the perception of the main objects for the prosthetic wearers. Thus, in this paper, we introduced a dual-threshold and multiregion connectivity analysis method for the saliency map segmentation. Compared with the single-threshold method, the new method improves the accuracy of the segmented objects [20]. The method first uses a dual-threshold to segment the saliency maps and then performs a connectivity analysis. Then, a morphology is adopted to eliminate small regions and isolated pixels. The main steps of the improved method are summarized as Algorithm 1.

### 2.3. Edge Detection

For the edge detection step, a multiscale Sobel operator is adopted to extract edge feature. Sobel operator is a kind of first-order differential edge detection operator. It is performed by extracting the gradient of the image. The gradient magnitude and direction reflect the edge strength and direction. The first-order differential operator with the scale *σ* is used to extract the image gradient ∇*f*. The image gradient is defined as

(5) $$\begin{array}{c} \nabla f=\left[\begin{array}{c} G_{x}\\ G_{y} \end{array}\right]=\left[\begin{array}{c} \frac{\partial f}{\partial x}\\ \frac{\partial f}{\partial y} \end{array}\right], \end{array}$$

where *G*_*x*_ is image horizontal gradient and *G*_*y*_ is the vertical gradient. The magnitude and direction of gradient are defined as (6). The edge is obtained by the comparison between the gradient magnitude and the set threshold

(6) ∇f=mag∇f=Gx2+Gy21/2,α=arctan⁡GxGy.

The edge is obtained by the comparison between the gradient magnitude and the set threshold. However, some local changes of the image cannot be detected in a Sobel operator with single scale. Small scale operators can locate the edge with more accuracy, but it is sensitive to noise. Large scale operators are the opposite. In order to solve the problems, this paper presents a multiscale Sobel operator to detect the edge features. It computes the edge magnitude with Sobel operators at different scales. The final edge magnitude is the geometric magnitude mean at different scales. The edge magnitudes mappings are ∇*f*_*N*_ at *k* scales (*N*_*k*_ = 3,7); the proposed multiscale edge strength is shown in

(7) $$\begin{array}{c} \nabla \tilde{f}={\left(\;\prod_{k}\nabla f_{N_{k}}\;\right)}^{1/k}. \end{array}$$

**Figure 3:**
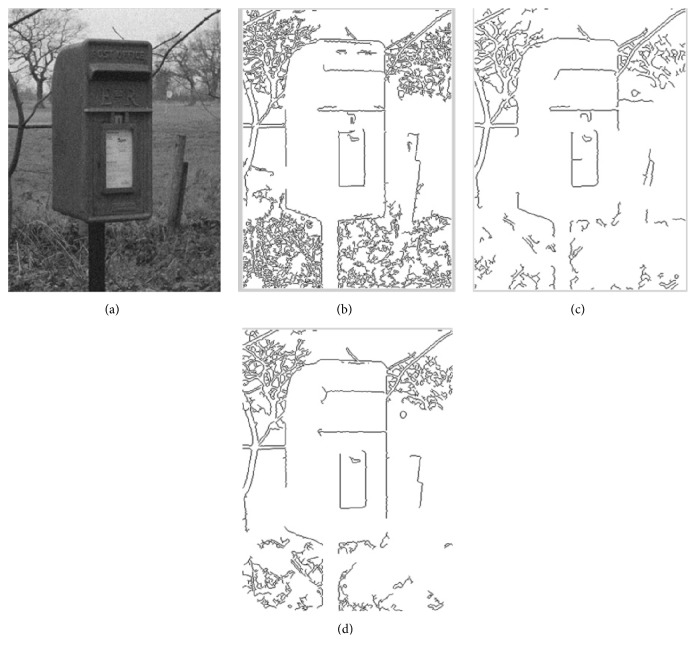
Results of edge detection under different scales. (a) Image with white Gaussian noise, (b) Sobel edge detection with scale *N*_*k*_ = 3, (c) Sobel edge detection with scale *N*_*k*_ = 7, and (d) fused edge detection with multiscales.

We set the scale *k* = 2, the size of Sobel operator *N*_*k*_ = 3,7, and the original image is with white Gaussian white noise.  Figure 3 shows the results of edge detection with different scales. In Figures 3(b) and 3(d), the result of multiscale edge detection contains less spurious edges and texture features; this means that the multiscale method has good noise robustness. In Figures 3(c) and 3(d), multiscale method can detect more accurate edges and the edge detection accuracy is better. In summary, the proposed edge detection method has better performance.

### 2.4. “FEBR” Processing Strategy

The “FEBR” processing strategy is used to enhance the contrast between the main object and the background under simulated prosthetic vision with low resolution. The “FEBR” processing strategy is able to increase the object recognition rate. For the foreground image *f*_F_(*x*, *y*), edge detection is carried out to enhance the contour information. In this paper, as shown in (8), we applied a multiscale edge detection technique as described in Section 2.3 for the foreground image. For the background image *f*_B_(*x*, *y*), as shown in (9), the gray values are linearly reduced to its half, so that the background gray values are set to the range of 0–127. Finally, the edge detection foreground *g*_F_(*x*, *y*) and reduced background *g*_B_(*x*, *y*) are fused as *g*(*x*, *y*), as shown in (10)

(8) gFx,y=EfFx,y,

(9) gBx,y=αfBx,y=fBx,y2,

(10) gx,y=gFx,y+gBx,y.

### 2.5. “FZE” Processing Strategy

**Figure 4:**
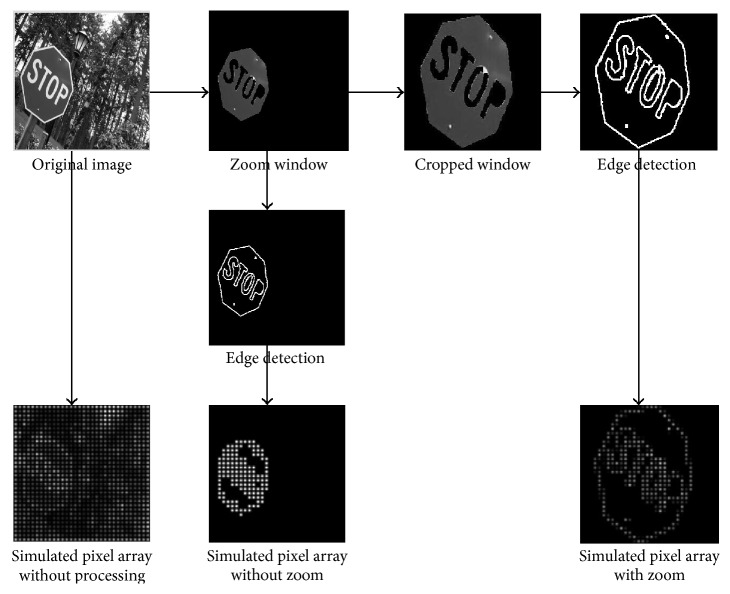
Flowchart of the FZE processing strategy.

The “FZE” processing strategy is used to detect and enhance the edge feature of the foreground objects to increase the recognition rate. Before performing edge detection, we add a zoom step to increase the size of the target and make it occupy the entire visual field. The zoom step takes the minimum sized box containing the foreground pixels as the zoom window. Then, the zoom window is cropped to the size of the final presented image. In Figure 4, an image sample “traffic signs” is used to illustrate the main step of FZE processing strategy. It shows that the details in the zoomed image are clearer and the contour is more significant.

### 2.6. Phosphene Model

To simulate the real visual perception, this paper uses a phosphene model based on a Gaussian distribution [21]. The image is divided into regions with fixed size and pixels in the regions are combined. The mean gray value of the pixels in the region is used as the central luminance value of the Gaussian points. The Gaussian curve is the luminance distribution of simulated phosphenes. This model is described in

(11) $$\begin{array}{c} I\left(\;x,y\;\right)=A\left(\;u_{x},u_{y}\;\right)•G\left(x,y\right), \end{array}$$

where *A*(*u*_*x*_, *u*_*y*_) is the gray value of the stimulated pixels and *G*(*x*, *y*) represents the Gaussian distribution function, as is shown in

(12) $$\begin{array}{c} G\left(\;x,y\;\right)=\frac{1}{2\pi \sigma^{2}}e^{-\left({\left(x-u_{x}\right)}^{2}+{\left(x-u_{y}\right)}^{2}\right)/2\sigma^{2}}. \end{array}$$

**Figure 5:**
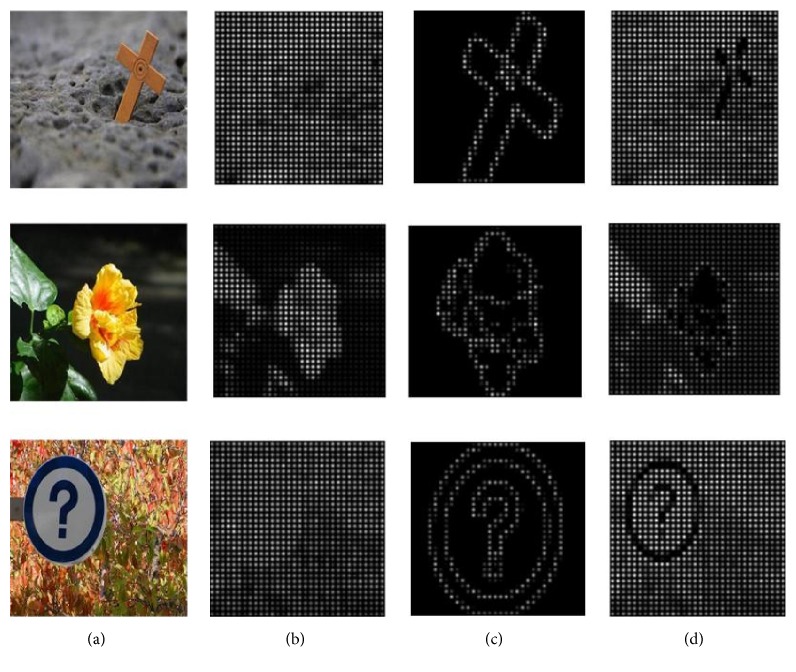
Results under different strategies after LRG: (a) original image, (b) image processed after LRG without optimization, (c) image processed after LRG under the strategy FZE, and (d) image processed after LRG under the strategy FEBR.

The images processed by the phosphene model correspond to the actual electrode array of visual prosthesis. This process is called “Lowering Resolution with Gaussian (LRG) dots.” After LRG pixelization, the images processed by “FEBR” and “FZE” were converted to 6 different phosphene resolutions.  Figure 5 shows the image processed by “FEBR” and “FZE” after LRG with the resolution 32*∗*32.

## 3. Psychological Experiment and Analysis of Results

### 3.1. Results of Salient Object Detection Model

**Figure 6:**
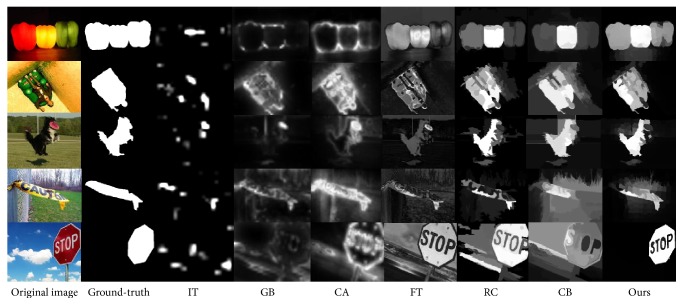
Comparison of different saliency detection algorithms.

**Figure 7:**
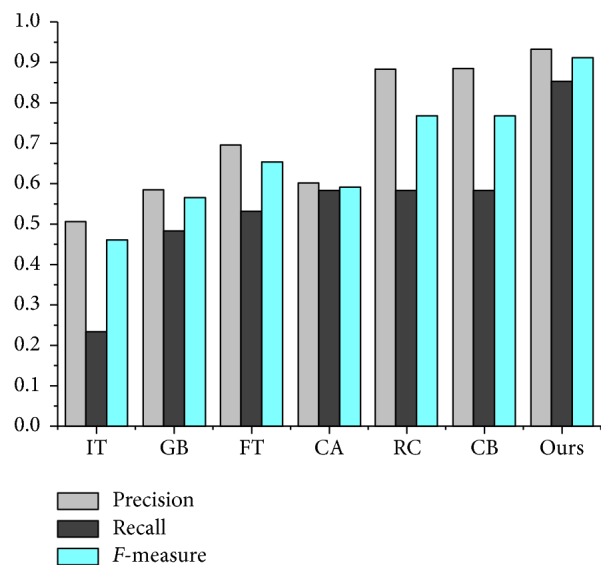
Comparison of different saliency detection algorithms.

This paper adopts a two-stage saliency detection scheme based on the manifold ranking. It makes full use of the image's intrinsic manifold structure, which can be effectively used to highlight the target uniformly as well as compressing the background. In order to illustrate the advantages of the model, we compare this algorithm with other saliency detection algorithms such as IT, GB, FT, CA, RC, and CB [15, 16, 20, 22–24].  Figure 6 shows the saliency map detected by the different algorithms. From the analysis of the data, we can conclude that the saliency map detected by the manifold ranking model can highlight the target object evenly and maintain a good boundary. Figure 4 shows the three indexes of precision, recall, and *F*-measure computed under the MSRA-1000 image database. The relation of the three indexes is shown in (13), where *β* = 0.3. In Figure 7, it demonstrates that the saliency detection model we adopted has the highest precision, recall, and *F*-measure. In Table 1, we compare the average time taken by each method on Intel Core 2.8 GHz machine with 2 GB RAM. It showed that our method has taken the lowest average time and is sufficient for real-time applications. The results showed that our method can produce superior saliency map with the real-time compared with other algorithms

(13) $$\begin{array}{c} F\text{-measure}=\frac{\left(1+\beta^{2}\right)\;\text{precision}\times \text{recall}}{\beta^{2}\text{ precision}+\text{recall}}. \end{array}$$

### 3.2. Results of the Saliency Map Segmentation

**Figure 8:**
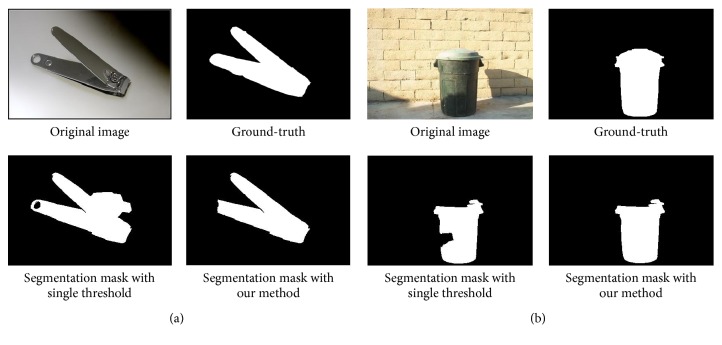
Comparison of different saliency segmentation method: (a) example image “nail clippers” and (b) example image “dust bin.”

The salient object mask is segmented using the adaptive double-threshold method. In order to evaluate the performance of the segmentation method, we compare the mask data of salient object with the ground-truth. In Figure 8, some incorrect pixels contained in the salient object mask that segmented by traditional segmentation method. Compared with the traditional salient object mask, in our method the incorrect pixels are rectified using the adaptive double-threshold segmentation method. This improved segmentation method is useful for rectifying some incorrect classified pixels, but it cannot correct every mistaken pixel. In the final segment mask, compared with the ground-truth we are still left with some incorrect pixels and regions.

### 3.3. Psychological Experiment

In order to verify the recognition rate of the objects in real-life scenes using the processing strategies proposed in this paper, we designed a psychophysical experiment for visual prosthesis. The materials used in the experiment were images selected from our daily life and were essential in our daily life. The visual field was 20 that simulates the prosthesis device. The resolution of every image was normalized to 320*∗*320. In order to avoid the influence of resolutions, the visual field of the main object in the image covered the angle of 12–14°. The subjects who participated in the experiment were 16 volunteers chosen from Xi'an University of Technology. They (8 males and 8 females) were aged from 20 to 25 years. They were all with normal or corrected visual acuity. The experiment was performed in accordance with the Declaration of Helsinki.

Subjects were seated 60 cm in front of a 21-inche LCD monitor (Lenovo INC, Beijing, 1280*∗*1024 resolution, 26 diagonal visual field). The images were randomly displayed on the center of the monitor. The experimental process was controlled by the psychological toolbox software “PsychToolbox-3” [25]. Before the start of the experiment, the subjects were provided with a list of the experimental objects which would be shown to them. This helped the subjects familiarize themselves with the upcoming objects and the experimental protocol. During the experiment, the images were divided into two groups (“FZE” and “FEBR”). Each group of images was randomly presented to the participants.

The recognition score (RS) was used to quantify the recognition results. If the subjects were able to correctly recognize the objects and give the right name, RS was set to 2. If the subjects could not correctly name the object and they could describe the shape or specific features of objects, RS was set to 1. Otherwise, the RS was set to 0. As shown in (14), the values of RS were normalized to the recognition accuracy (RA) under different processing strategies. The Statistical Product and Service Solutions (SPSS) for Windows (SPSS Inc.) software is used to perform statistical analysis. The results are expressed in the form of mean ± SEM (standard error of mean). A two-factor analysis of variance (ANOVA) was adopted as the metric to evaluate the effect of statistical significance of the resolution and the image processing strategies

(14) $$\begin{array}{c} RA=\frac{RS}{2}\times 100\%. \end{array}$$

### 3.4. Results of the Object Recognition

**Figure 9:**
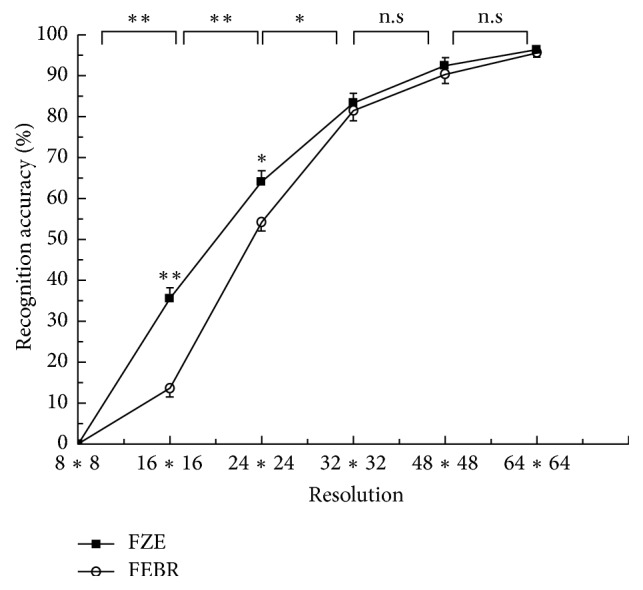
Comparison of the object recognition rate under different resolutions (^*∗*^*P* < 0.05, ^*∗∗*^*P* < 0.01).

In this experiment, the accuracy of the object recognition task was evaluated under 6 different resolutions.  Table 2 and Figure 9 show the RA scores with two processing strategies under different resolutions.

The image resolution has a statistically significant effect on the RA scores (*F* = 189.112, *P* < 0.05) for the task of object recognition. The RA scores were close to 0 when the resolution was 8*∗*8. When the resolution was increased to 16*∗*16, noticeable differences were shown in two processing strategies (*F* = 93.664, *P* < 0.05). When the resolution increased to 24*∗*24, a great increase in the recognition accuracy was observed. A relatively slow increase in the recognition accuracy from 80% to 96% was observed when the resolution was increased from 32*∗*32 to 64*∗*64. On the whole, the FEBR strategy demonstrated higher recognition accuracy compared with the FZE strategy.

## 4. Discussion

Introduction of a certain image processing technique is considered to be an effective method to optimize the presentation of the visual information in visual prosthesis. Some basic image processing methods such as edge detection and contrast enhancement have been applied to some retinal prosthesis systems. In this paper, we demonstrate that the introduction of a saliency detection model based on manifold ranking and a segmentation technique based on the multithreshold and connectivity analysis have significant effects on the segmentation of main objects in daily scenes. Two processing strategies are proposed to optimize the image presentation. These strategies can help extract and present the main information from real-life scenes and help a blind person successfully complete the tasks of perception and recognition of objects in a given scene. Through psychophysical experiments, we show that the proposed image processing methods can significantly improve the ability of a person's object location and recognition.

### 4.1. Effect of Salient Object Extraction

Automatic detection and extraction of main objects in a scene are a key step in the processing strategy. The proposed saliency detection model and the segmentation technique have the ability to segment objects in 60 experimental materials with an accuracy of 90%.

The validity of the object segmentation will affect further processing for object enhancement and the performance of the task of perceiving and recognizing of objects for prosthetic users. The recognition performance in the two processing strategies is analyzed to show that segmentation significantly affects the recognition rate. Segmentation closer to the real scene improves the accuracy of the object recognition. This makes it very clear that our proposed method cannot be similar to the function of human eyes which can extract complete objects from complex scenes. The objects extracted using our proposed method will always either miss some part of the content or contain some unnecessary background information. According to prosthesis research, edge information has a significant influence on the recognition of objects. If the edge information of the extracted object is not well preserved, the final recognition performance will be relatively poor.

The most important factor affecting the accuracy of object extraction is the generation of the saliency map. It is also a technical challenge in the research area of saliency region segmentation. The main objects are not marked by adequate salient points in poorly segmented materials. Although the computational model based on manifold ranking provides huge benefit for saliency extraction, some objects in the image cannot get enough large area of interest. More efficient saliency models, ROI definitions, and segmentation methods need to be adopted in future research to achieve more accurate objects extraction from the daily scenes.

### 4.2. Effect of Image Processing Strategy

Obtaining objects from real-life scenarios can be used to enhance the presentation of the objects to the blind people. Edge information is an important object feature and is the main factor which affects the performance of object recognition during low resolution. To enhance the foreground information, the presentation strategy uses foreground zooming and keeps the edge information. Experimental results in simulated prosthetic vision show that foreground zooming and edge detection can effectively improve the recognition accuracy of subjects in the good segmentation. Although the individual recognition results are not good in poor segmentation, they do not affect the overall performance of the image recognition task. Due to the zoomed foreground, this approach presents more edge detail information to the user than the direct edge detection approach. We report that the foreground zooming strategy in this study has the highest recognition results. Based on this, in the current stage of retinal prosthesis systems with less than 1000 electrodes, foreground zooming is more suitable for visual presentation. By enhancing the foreground information, not only will the number of correctly named objects be greatly improved, but also the subjects can describe the objects in the images more accurately. Although the two processing methods remove certain background information, they also reduce the influence of scene illumination to highlight the main information in the scene. This is highly significant to obtain better visual task performance under limited visual perceptions.

The results of different image analyses show that different subjects have different recognition abilities. With the same segmentation, objects with simple shape are relatively hard to recognize. But the subjects are able to accurately describe the image after enhanced expression. The recognition rate of the objects with complex contour information is much higher than objects with simple shape. Zhao et al. [7] made similar observations during their experiment. In this study, we suggest that this is mainly due to the lack of features contributing to the recognition. We also suggest that regional features should be enhanced to make up for this phenomenon. This study also shows that the edge contour information is very important for object recognition in low resolution.

### 4.3. Effect of Image Resolution

The results demonstrate that the recognition accuracy is significantly affected by the resolution. When the resolution increased from 16*∗*16 to 24*∗*24, the RA scores increase greatly and reach values higher than 50%. When the resolution reaches 32*∗*32, it performs well for the task of object recognition without any prior information. We can therefore conclude that higher resolutions images have positive effects on the task of visual recognition. But we also observed that increasing the resolution cannot compensate for the huge information loss caused by pixelization.

### 4.4. Effect of Phosphene Model

In this paper, we simulate the visual percepts with the phosphene model with Gaussian distributions. In fact, current visual percepts provided by visual prosthesis contain multiple factors that would influence recognition, including distortion, dropout, and shape irregularity. Insights from psychological experiments and theoretical considerations suggest that the interaction between implant electronics and the underlying neurophysiology of the retina will result in spatiotemporal distortions that include visual “comets” in epiretinal prostheses and motion streaks in optogenetic devices [26]. Nanduri et al. [27] demonstrated that a significant change will be caused in size and shape of phosphenes with the increasing stimulation amplitude. The perpetual distortions lead to a lack of retinotopic correspondence between the stimulation site and the perceived location of the phosphenes in visual space [28–30]. Lu et al. [31] showed that the recognition linearly decreased with an increase of distortion level, meaning that the distortion had a serious impact on the object recognition. From another perspective, the results indicated that recognition accuracy could be improved for prosthesis wears by distortion correction. These factors will be considered in future studies for more realistic practices.

## 5. Conclusion

In this paper, different visual information processing strategies were explored to optimize the presentation of visual information under simulated prosthetic vision. The saliency detection model is introduced to detect salient objects in real-life scenes. A multithreshold method is proposed to improve the foreground segmentation. Two processing strategies are carried out to optimize the presentation of visual information. Experimental results demonstrate that the two strategies significantly improve the visual perception and recognition rate of objects under low resolution. This work can be used to help blind people to significantly improve their ability to adapt to the surrounding environment.

## Figures and Tables

**Algorithm 1:**
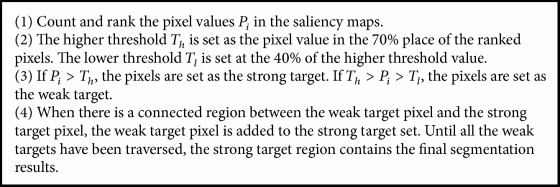
The main steps of the image segmentation algorithm.

**Table 1:** Average time taken to compute saliency map for images in MSRA-1000 database.

Method	IT	GB	FT	CA	RC	CB	Ours
Time (s)	0.246	1.614	0.102	38.896	0.154	2.146	0.091
Code type	MATLAB	MATLAB	C++	MATLAB	C++	MATLAB	MATLAB

**Table 2:** Results of the object recognition rate under different resolutions.

Strategy	Resolution					
	8*∗*8	16*∗*16	24*∗*24	32*∗*32	48*∗*48	64*∗*64
FZE	0.00 ± 0.00	35.46 ± 2.59	64.13 ± 2.58	83.29 ± 2.38	92.45 ± 1.83	96.32 ± 1.12
FEBR	0.00 ± 0.00	13.57 ± 2.13	54.14 ± 2.23	81.39 ± 2.42	90.26 ± 2.19	95.56 ± 1.09

## Abbreviations

FZE: Foreground zooming and edge detection
FEBR: Foreground Edge Detection and Background Reduction
LRG: Lowering Resolution with Gaussian dot
ROI: Region of Interest.
